# Effects of the Visual Character of Transitional Spaces on Human Stress Recovery in a Virtual Reality Environment

**DOI:** 10.3390/ijerph192013143

**Published:** 2022-10-12

**Authors:** Zhixian Li, Xiaoran Huang, Marcus White

**Affiliations:** 1 College of Environmental Design, University of California, Berkeley, CA 94720, USA; 2 School of Architecture and Art, North China University of Technology, Beijing 100144, China; 3 Centre for Design Innovation, Swinburne University of Technology, Hawthorn, VIC 3122, Australia

**Keywords:** linear environment, curved environment, human stress recovery, transitional space, virtual reality (VR)

## Abstract

As people’s levels of stress increase with the complexity of contemporary urban life, the stress healing agenda in built environments has become more critical than ever. Previous research has demonstrated that linear and nonlinear shapes in the environment have an impact on human stress recovery. However, to date, most studies have focused on indoor and outdoor spaces, while research on transitional spaces is still limited. Transitional spaces connect the interior with the exterior and are ubiquitous in the city, such as plazas, open cafes, and urban corridors. We hypothesize that curved and linear environments affect human stress recovery differently in transitional spaces. To test this hypothesis, virtual reality (VR) technology and experiments were conducted with 40 participants. At the end of the Trier Social Stress Test (TSST), participants were randomly assigned to four VR environments to test which environment is more effective in stress recovery for humans. Participants’ physiological data, including heart rate and blood pressure, were measured by bio-monitoring sensors. The psychological data were tested by the State-Trait Anxiety Inventory (STAI). In general, the resulting data indicate that the curved environment is more effective than the linear environment for the recovery of human stress in transitional spaces.

## 1. Introduction

With the continued development of the economy and society, city life is generally associated with long working hours, heavy workloads, tight deadlines and unsatisfactory working conditions [1]. At the same time, the risk of mental health problems is increasing among people who are exposed to psychosocial work stress in the work environment [2,3]. Stress is a widespread disorder that significantly limits psychosocial function and impairs quality of life. According to the World Health Organization, stress has become a 21st-century health epidemic [4]. Moreover, mental illness has become the largest epidemic in the world [5]. One in five young people in the United States (approximately 46.6 million) suffers from mental illness due to the stress of work, school, and social relationships [6]. High levels of stress often lead to a range of health disorders, such as obesity, diabetes and cardiometabolic complications [7,8]. Human health and wellbeing have been impacted by their living environment [9], and the spatial environment is one of the influencing factors of human stress [10,11,12], which can be alleviated with some appropriate spatial design [13]. In order to reduce the negative impact of these stresses on humans, more effective measures to improve human stress are necessary [14].

Some research has suggested that certain kinds of environments are correlated with improved mental health and a reduced risk of certain types of mental illness [15]. In recent years, scholars have increasingly studied the spatial environment and the influencing factors in the spatial environment that contribute to human stress recovery [16,17,18]. Some research has indicated that there is a diversity of factors that affect human stress in indoor and outdoor spaces such as lighting, outdoor landscaping, noise, temperature, and humidity [19,20,21,22,23]. Moreover, some researchers have investigated the impact of geometric forms in the environment on human perception, for example, linear and curved factors. Curved forms occupy a unique position in the tradition of Western philosophical, psychological and evolutionary aesthetic thought [24,25]. Some scholars have researched the effects of linear and curved environments on human stress with neuroscience experiments showing that people experience less stress in the curved form than in the linear form [26]. In the research of interior spaces, Carreiro et al. recruited 32 test participants to evaluate 18 VR architectural structures based on aesthetic judgments; their study showed that architecture with rounded, curved elements was interpreted as more pleasant and likeable than sharp, straight elements [27]. The rectilinear or curvilinear forms of furniture in an interior environment can also have a differential effect on human emotions, with curved environments causing more pleasant emotions (e.g., feelings of relaxation, peace and calm) than linear environments [28]. Additionally, some studies have shown that the participants’ professions have an effect on preference and cognition [29,30,31]. Shemesh et al. indicated that participants with a non-design background felt better in environments with curved edges and rounded contours, while participants with a background in design preferred linear environments [32]. Furthermore, some researchers have studied the effects of linear and curved factors in outdoor spaces on human emotions and stress. Several studies have shown that soft, curved edges are considered visually appealing and pleasing to humans in landscape design [33]. The public prefers landscape objects with curved edges rather than angular edges, and feels relaxed in curved environments [34]. Building facades also generally provide different visual stimulus and sensation [35], and studies on the preference of building facades in urban spaces have shown that curved facades usually provide a better sensation [36]. A research study conducted on university students’ preference for campus environment showed that informal design and curved paths are more effective in reducing students’ stress [37]. The research on preference for riparian buffers found that people prefer curved riverbeds to straight riverbeds outdoors [38]. 

**Figure 1:**
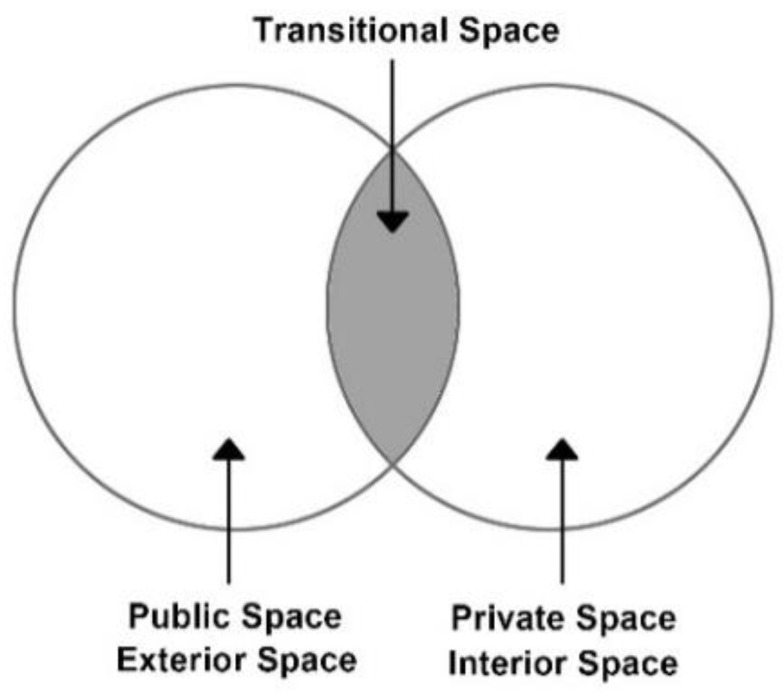
The transitional space is a buffer zone between the indoor space and the outdoor space.

**Figure 2:**
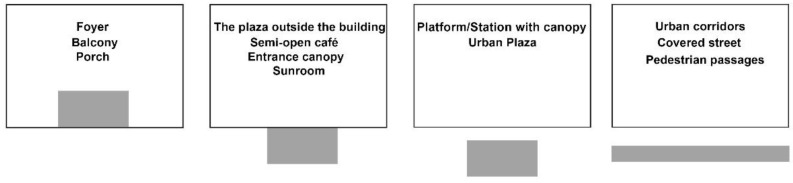
Some spaces that have been defined as transitional spaces in previous research.

However, most existing studies about human stress recovery have focused specifically on indoor, or entirely outdoor spaces. In addition to indoor and outdoor spaces, there is also a “third space”, that is, the transitional space (Figure 1). These transitional spaces (intermediate or third zones) are experiential spaces between internal and external spaces [39,40]. Transitional spaces are defined as the in-between space of private or public domains that act as both buffer spaces and physical connectors, such as atriums, plazas, urban corridors, gathering spaces, passages, courtyards, and stairwells [41,42,43]. Figure 2 provides a summary of some of examples that have been defined as transition spaces in the previous literature [44,45,46]. There remains a research gap in terms of the impact of transitional spaces on human stress. Transitional spaces are embedded in people’s daily lives, and almost everyone passes through them every day. Therefore, it is meaningful to improve the stress recovery rate on people in transitional spaces. 

Therefore, to contribute to the literature on the restorative impact of friendly transitional spaces, the objectives of this study are as follows: (1) to compare whether linear or curved environments in transitional spaces have a more positive effect on human stress recovery by combining physiological indicators (HR, DBP, SBP) and psychological indicators (STAI); (2) to quantify the positive/negative effects of linear and curved environments in transitional spaces on human stress recovery. 

## 2. Methods

### 2.1. Research Process

This study focuses on the effects of curved and linear environments on the stress recovery of humans in transitional spaces. Firstly, it is assumed that curved and linear spaces have different stress recovery effects for humans in the transitional space. The Trier Social Stress Test (TSST) has been proven to induce moderate psychological stress in which participants must present a speech (5 min) and perform mental arithmetic (5 min) in front of an audience [47]. Thus, a 10 min TSST experiment was conducted with the participants to increase the participants’ stress level. Then, participants were randomly entered into the VR scenario separately to test the effect of linear and curved environments in the transitional space on human stress recovery. During this process physiological, data are collected, including heart rate (HR) and blood pressure (BP). The blood pressure includes the systolic blood pressure (SBP) and the diastolic blood pressure (DBP). Psychological data are collected through the State-Trait Anxiety Inventory (STAI). Finally, data analysis is conducted by statistical product and service solutions (SPSS). 

### 2.2. Research Tools

This research uses a combination of virtual reality (VR), heart rate (HR), blood pressure (BP), and State-Trait Anxiety Inventory (STAI) to establish a systematic process to assess human emotions and to contribute to the harmonious development of humans and their environments.

Virtual reality (VR) technology, with its immersive displays, allows for the rapid construction of a model and simulation of multiple spatial and environmental scenarios. VR is also often used as an experimental tool in the research of human emotions. Annerstedt et al. researched the effect of natural sounds on stress recovery, and the result showed that a VR scenario with natural sounds increased the stress recovery rate in humans [48]. By setting up different scenarios in VR and conducting experiments, Valtchanov identified a significant improvement in human stress in natural environments compared to urban and geometric environments [49]. Yin et al. recruited 28 participants to experience three versions of a pro-biological design in an open and closed office space simulated in VR [50]. The results suggest that the pro-biological intervention can help reduce stress and increase creativity. In addition, previous research has also shown that human psychological perception and physiological reactions are similar in VR scenarios and real scenarios [51]. Moreover, virtual reality technology helped to eliminate some of the interference factors in the experiment, such as auditory perception, olfactory system, and interaction with surrounding people. Thus, VR has been selected in this research.

In recent years, many scholars have used bio-monitoring sensors to test human heart rate and blood pressure to quantify the relationship between human perception and the built environment. Blood pressure and heart rate are often used as indicators of a human stress level [52,53]. Different basic emotions produce different physiological effects on the body. Surprise, fear, anger, pleasure and disgust can be reflected by variations in blood pressure [54]. In this research, heart rate and blood pressure were used to measure physiological data of stress recovery in humans.

The psychological indicators of the participants were measured by the six-item short-form of State-Trait Anxiety Inventory (STAI), which includes six questions [55]. It was developed by Marteau and Bekker from Spielberger’s full version that includes 20 questions on state anxiety and was proven to be similar to the scores of the full version [55,56]. The short version of the STAI includes three anxiety-positive questions (e.g., “I feel upset”; “I am nervous”) and three anxiety-negative questions (e.g., “I feel pleasant”; “I am relaxed”). Each question was scored with a four-point scale (e.g., “1 = not at all”, “2 = somewhat”, “3 = moderately” and “4 = very much”) in which the three positively worded items were reverse coded after the test, and the mean score of the six questions was calculated for each participant. A higher STAI score indicates higher anxiety level, and conversely, a lower score indicates lower anxiety level. Therefore, in this study, heart rate (HR) and blood pressure (BP) were used to determine response to stimulus in combination with the State-Anxiety Inventory (STAI) to evaluate the self-reported emotional state of the participant.

## 3. Experimental Design

### 3.1. Research Population

We recruited 40 healthy adults with an age range limited to 18–35 years to participate in this study through campus announcements and a social networking platform (WeChat). The research objectives were not disclosed during the recruitment period to reduce the potential for self-selection bias. Participants voluntarily enrolled in the experiment. Through a pre-screening process, we then excluded participants who claimed to have previously taken stress recovery medications or treatments. All participants had normal or corrected vision and were without mental impairment or disease, and they signed an informed consent form for the study prior to the experiment.

### 3.2. Research Design

We used a between-subjects design in this experiment. There are two reasons for this. Firstly, each participant only experienced the TSST once to ensure the best stress-increasing effect and avoid the potential for residual effects. Secondly, in order to reduce the negative effects of wearing the VR device on the participants, such as nausea and headache, the between-subjects design can reduce the wearing time of the VR Environment simulation.

**Figure 3:**
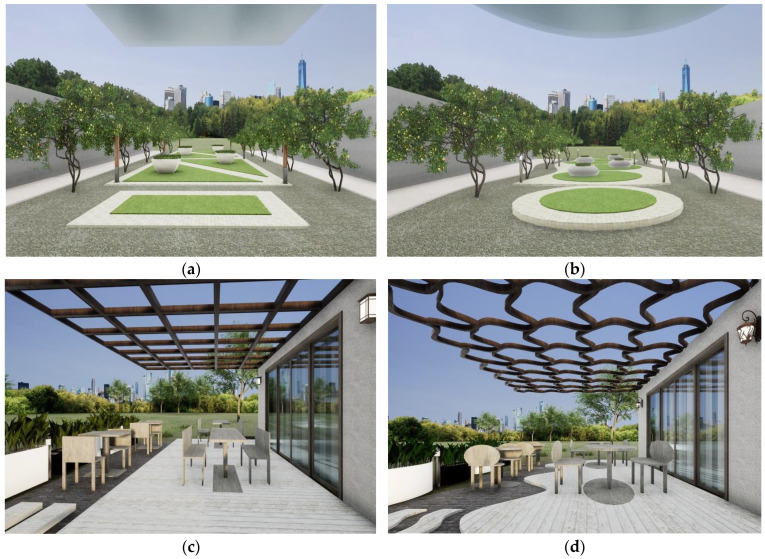
The layout of the four virtual reality transitional spaces. Note: A _curved_ and A _linear_ is café space, B _curved_ and B _linear_ is plaza space. (**a**) A _curved_ environment; (**b**) A _linear_ environment; (**c**) B _curved_ environment; (**d**) B _linear_ environment.

To test the participants’ reactions to linear and curved environments in different transitional spaces, models were created in Rhinoceros 3D and are presented in real time in the experiment using an industry-level immersive architectural visualization software Sheencity MARS (version 3.05.84.60, Sheencity, Chongqing, China) and VR heat mounted equipment (oculus quest 2). Two transitional spaces were chosen: a café and a plaza, which are two typical transitional spaces in everyday life. In the plaza space, a linear environment (A _linear_) and a curved environment (A _curved_) were created; similarly, in the café space, a linear environment (B _linear_) and a curved environment (B _curved_) were created (Figure 3). This design aimed to set up multiple scenarios to make the results more generalizable. Linear is defined as “of or in lines” in the Oxford Learner’s dictionaries. Curve is defined as “having a round shape” in the Oxford Learner’s dictionaries [57]. Linear environment means that the environment has more components in linear shape; curved environment means that the environment has more components in curved shape. The transitional space of the café space included overhead weather shelter, benches, tables, planters, flooring, and lights. In the transitional space of the plaza, the space included overhead shelter, flowerbeds, and landscape treatment. In the linear environment, the geometry of above environment components was controlled to be linear; similarly, in the curved environment, the geometry of above environment components was controlled to be curved.

### 3.3. Outcome Measures

This study measured the participants’ HR, SBP and DBP to quantitatively assess the effects of the built environment on human stress. Specifically, participants wore a Polar H10 heart rate monitoring chest belt to obtain primary HR data, with HR output at a frequency of once per second. SBP and DBP data were collected by an Omron HEM-7121 electronic blood pressure monitor and tested three times: at baseline, after the TSST test (i.e., before recovery) and after the 5 min VR experiment (i.e., after recovery). In addition to the above physiological effects, the psychological effects were collected by the State-Anxiety Inventory (STAI) using the six-item short-form of State-Trait Anxiety Inventory (STAI) to measure participants’ stress levels.

### 3.4. Experimental Procedure

**Figure 4:**
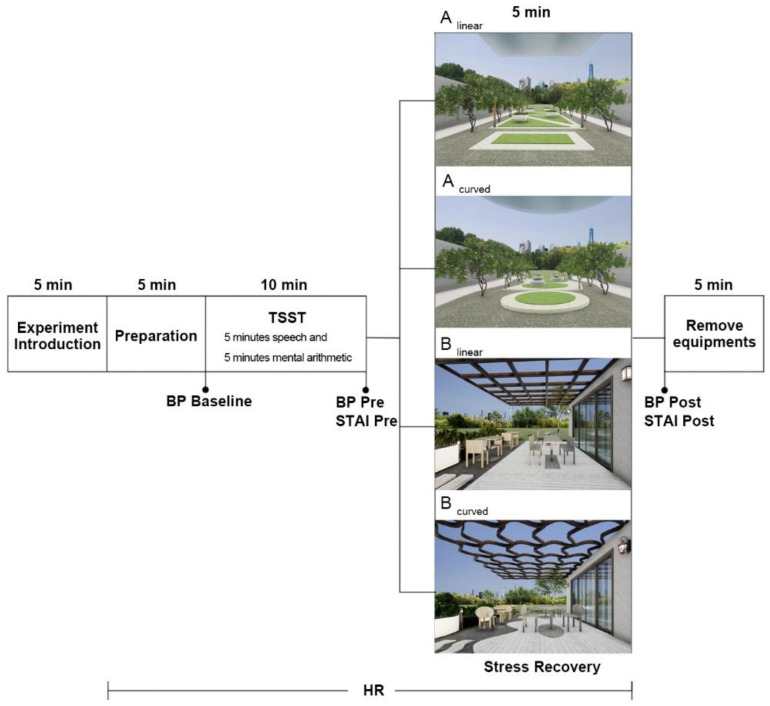
Experiment procedure.

To ensure scientific and accurate data processing, the experiment was conducted in the following steps, with the entire experiment taking approximately 35 min (Figure 4). After participants entered the experiment, they were asked to perform a 5 min preparation period, which was designed to reduce heart rate fluctuations caused by the participants’ discomfort with the environment. After the preparation period, the researcher assisted the participants in the fitting of the HR and BP measurement devices and started the baseline measurements. Subsequently, a stress induction experiment (TSST) was conducted, which consisted of a 5 min speech and a 5 min mental arithmetic. During the speech stage, the interviewee was required to deliver the interview speech without interruption, with five interviewers looking at the interviewee with serious expressions and telling the interviewee to continue when the interviewee pauses. In the mental arithmetic stage, the participant was asked to repeatedly subtract 13 from the number 1376; there was no paper or pencil, only mental arithmetic, and if the participant gave an incorrect answer, a stern alarm sounded, and the participant had to repeat the subtracting process. At the end of the induced stress experiment, participants were measured for blood pressure and STAI pre data prior to recovery. They were then randomly assigned to enter a VR virtual scene for a 5 min recovery experiment. According to previous studies [58,59], 5 min is sufficient to test the effect of stress recovery on participants. Therefore, participants were randomly assigned to enter one of the four virtual environments for a 5 min stress recovery period. At the end of the experiment, HR and STAI post phase tests were performed, after which participants were removed from the HR and BP measurement devices. HR was measured continuously during the experiment, BP was measured three times (baseline, pre-TSST, post-TSST) and STAI was measured twice (pre-TSST, post-TSST). Several studies have shown that indoor environmental quality (IEQ) significantly impacts building performance and occupant satisfaction [60], and therefore, IEQ was controlled for in this experiment. Room temperature and data such as PM_2.5_, temperature, relative humidity and CO_2_ were recorded using monitoring equipment, and the IEQ indicator was measured every 5 min.

## 4. Data Statistics

All 40 participants completed the study. The participants were divided into four groups according to four scenarios, each with 10 participants, and the number of participants was the same for each scenario. Prior to testing, we ensured that the participants did not consume drugs including caffeine and alcohol that would affect the experiment’s results. All data were analyzed through IBM SPSS Statistics 25. To verify the validity of the experiment, an analysis of variance was performed to investigate whether participants’ physical data and psychological data at pre-TSST, TSST period and IEQ among four environments were similar or not. A paired samples *t* test was conducted using before and after TSST data to verify the validity of the stressors. The effect of recovery on the physiological indicators of the participants was evaluated using the difference between the pre-test and post-test as the amount of change, i.e., ∆D (∆D = pre-test − post-test). We grouped the ∆D of A _curved_ and A _linear_ as one pair and the ∆D of B _curved_ and B _linear_ as the other pair. If the difference between the two groups satisfied a normal distribution, a paired samples *t* test was used; if it did not satisfy a normal distribution, a non-parametric test was used. 

To investigate the relationship between continuous measurements (heart rate) and time, we used repeated-measures ANOVA to investigate their interaction. We used the five minutes of the recovery period as a within-subjects factor and the four scenarios as between-subjects factors with and an LSD (Fisher’s least significant difference) post hoc multiple comparisons. A statistical significance was identified using an alpha level of 0.05 for all data. This study used Cohen’s *d* as a characterization of the effect size. According to previous literature [61], we can interpret effect size using the negligible (<0.2), small (0.2–0.5), moderate (0.5–0.8), and large effect (>0.8).

## 5. Results and Analysis

### 5.1. Demographics

Table 1 presents the overall characteristics of the 40 participants. The same number of participants were tested in each scenario, with the same number of males and females in each, in order to control for the effect of gender on the results of the experiment. Participants reported no intake of stimulant foods such as caffeine or alcohol prior to testing. 

### 5.2. Experimental Randomization

To verify the validity of the experimental randomization, we conducted ANOVA on HR, SBP, DBP and STAI data in the pre-TSST and the post-TSST phase (Table 2). The ANOVA results indicated that there were no significant statistical differences in HR (*F* = 0.86, *p* = 0.47), SBP (*F* = 0.63, *p* = 0.60) and DBP (*F* = 0.86, *p* = 0.47) between participants in the different test scenarios during the pre-TSST period; similarly, HR (*F* = 0.62, *p* = 0.61), SBP (*F* = 0.04, *p* = 0.99), DBP (*F* = 0.61, *p* = 0.61) and STAI (*F* = 11.25, *p* = 0.002) were not significantly different between the test scenarios during the post-TSST period. Therefore, differences in the physiological indicators of the participants had no significant effect on the results of the experiment. In addition, an ANOVA was conducted on CO_2,_ PM_2.5_, temperature and humidity among the four environmental due to the potential effect of IEQ on human stress. The results showed no significant differences in CO_2_ (*F* = 0.55, *p* = 0.66), PM_2.5_ (*F* = 0.48, *p* = 0.70), temperature (*F* = 1.57, *p* = 0.25) and humidity (*F* = 0.77, *p* = 0.53) among the four scenarios. Therefore, there was no significant effect of IEQ on the experimental results.

To verify the effectiveness of the 10 min stressor (TSST) on increasing participants’ stress, we analyzed physiological data from participants before and during the TSST among four environments (Table 3). The results showed that SBP (*T* = −3.54, *p* = 0.001), HR (*T* = −15.59, *p* < 0.001) and DBP (*T* = −3.14, *p* = 0.003) before and during the TSST demonstrated significant differences. Thus, a 10 min stressor (TSST) was effective in increasing participant stress levels.

### 5.3. Stress Recovery Effects

**Figure 5:**
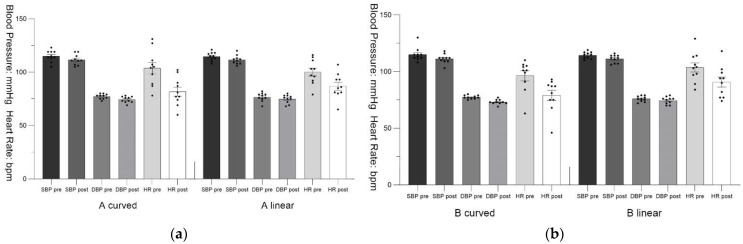
Pre-test and post-test of blood pressure and heart rate with the standard error mean bars: (**a**) Scenario A; (**b**) Scenario B.

**Figure 6:**
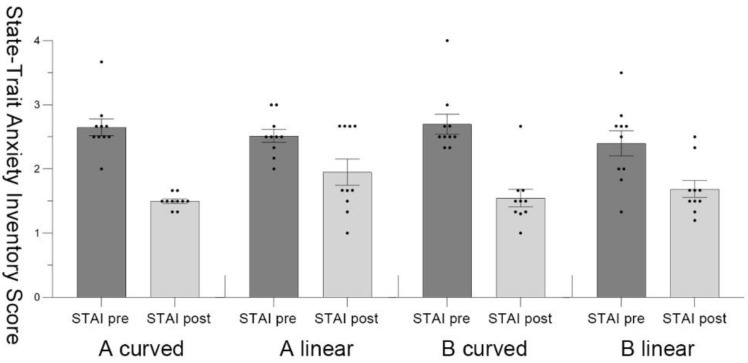
Pre-test and post-test of the State-Trait Anxiety Inventory (STAI) score with the standard error mean bars in scenario A and scenario B.

This research retains all the data from the measurements. The results showed some data with outliers. However, by comparing the pre-test and post-test data, a correlation was found between these data. Therefore, we presumed that these outliers are not due to errors in measurement, experimental design and recording. We chose to retain these data in order to ensure the authenticity of the results. Figure 5 and Figure 6 show the mean ± standard error for all data.

**Figure 7:**
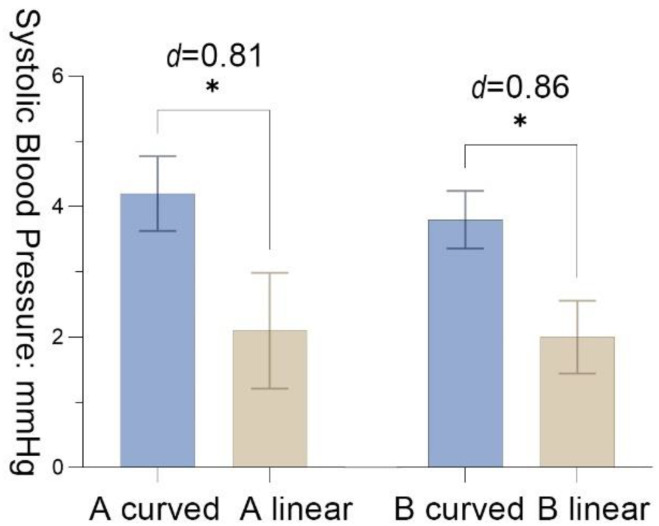
The results of systolic blood pressure with the standard error mean bars; significance levels: * *p* < 0.05; effect size (d): negligible (<0.2), small (0.2–0.5), moderate (0.5–0.8), and large effect (>0.8).

The paired samples *t* test for ∆D of SBP demonstrated that the mean value of A _curved_ after the recovery period was significantly higher than the mean value of A _linear_ (*M* = 2.10, *SD* = 2.60 *t* = 2.55, *p* = 0.03, Cohen’s *d* = 0.81). B _curved_ and B _linear_ are significantly different in recovery period, *M*_B_ = 1.80 (*SD* = 2.10, *t* = 2.71, *p* = 0.02, Cohen’s *d* = 0.86), which indicated that the recovery effect of B _Curved_ is better than B _linear_ (Figure 7). 

**Figure 8:**
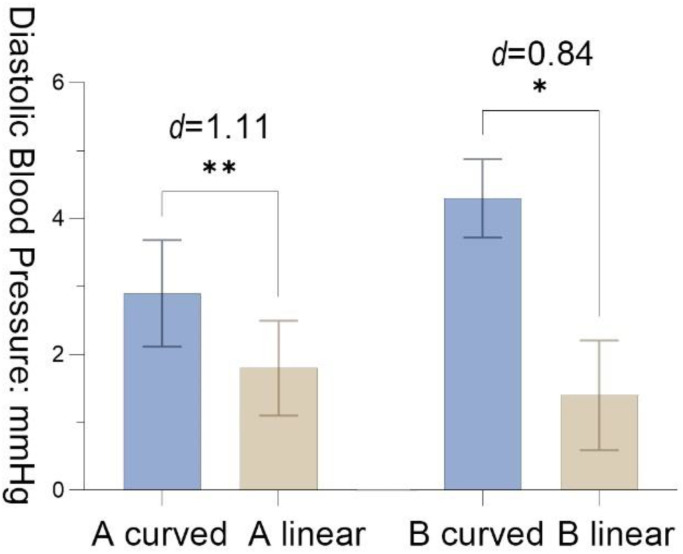
The results of diastolic blood pressure with the standard error mean bars; significance levels: * *p* < 0.05, ** *p* < 0.01; effect size (d): negligible (<0.2), small (0.2–0.5), moderate (0.5–0.8), and large effect (>0.8).

Physiological data for ∆D of DBP showed that the mean of A _curved_ was significantly higher than the mean of A _linear_ after the recovery period (*M* = 1.10, *SD* = 0.99, *t* = 3.50, *p* = 0.01, Cohen’s *d* = 1.11), demonstrating that A _curved_ was better at stress recovery for participants than A _linear_. B _curved_ and B _linear_ were significantly different in the recovery period, *M*_B_ = 3.3 (*SD* = 2.90, *t* = 2.66, *p* = 0.03, Cohen’s *d* = 0.84). Similarly, participants in the curved environment had better stress recovery effect in scenario B (Figure 8).

**Figure 9:**
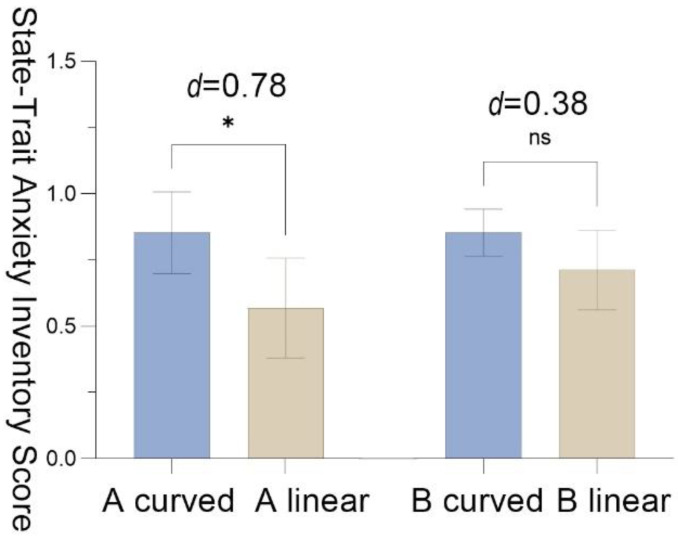
The results of the State-Trait Anxiety Inventory (STAI) score with the standard error mean bars; significance levels: ns *p* > 0.05, * *p* < 0.05; effect size (d): negligible (<0.2), small (0.2–0.5), moderate (0.5–0.8), and large effect (>0.8).

The result data of STAI indicated a statistically significant difference (*M* = 0.28, *SD* = 0.36, *t* = 2.47, *p* = 0.01, Cohen’s *d* = 0.78), which suggested that participants in A _curved_ had better stress recovery effect compared with participants in A _linear_. The participants’ stress recovery effect in B _curved_ was 0.14 scale higher than that in B _linear_, (*SD* = 0.37, *t* = 1.21, *p* = 0.26, Cohen’s *d* = 0.38) (Figure 9).

**Figure 10:**
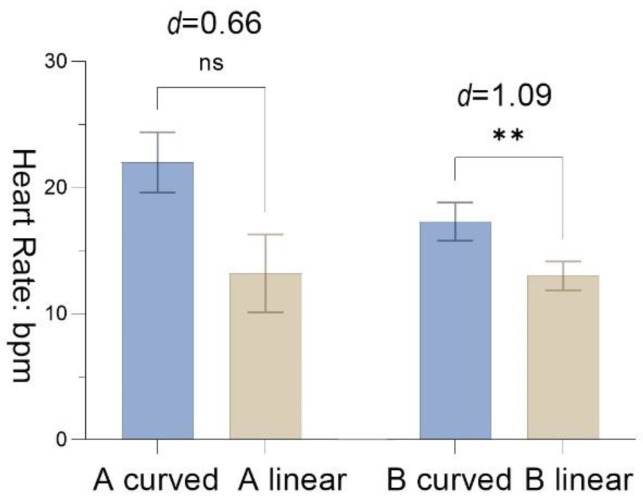
The results of heart rate with the standard error mean bars; significance levels: ns *p* > 0.05, ** *p* < 0.01; effect size (d): negligible (<0.2), small (0.2–0.5), moderate (0.5–0.8), and large effect (>0.8).

The ∆D for HR showed that participants had better recovery effect in A _curved_ than in A _linear_, with a difference of 8.80 scale (*SD* = 13.25, *t* = 2.47, *p* = 0.065, Cohen’s *d* = 0.66). Participants in B _curved_ had higher HR data than in B _linear_, with statistically significant differences (*M* = 4.30, *SD* = 3.95, *t* = 3.45, *p* = 0.01, Cohen’s *d* = 1.09), demonstrating that participants recovered better from stress in the B _curved_ (Figure 10).

### 5.4. Interaction of Continuous Measurement Data with Time

**Figure 11:**
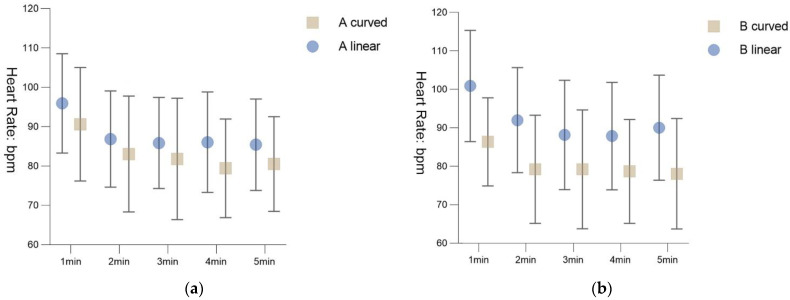
Mean and standard deviation of heart rate per minute: (**a**) Scenario A; (**b**) Scenario B.

The repeated measures ANOVA was performed to investigate the interaction between time and continuous measurement data (Figure 11). The Shapiro–Wilk test showed that the data in each group were normally distributed, and the Levene’s test showed that the homogeneity of variance was satisfied between the two groups at each time point. We used the five minutes of the recovery period as a within-subjects factor and the four scenarios as between-subjects factors. Mauchly’s sphericity test showed that the data of environment A does not meet the spherical hypothesis (*p* = 0.004). The time*group term in the multivariate test did not show a statistically significant difference (*df* = 4, *f*_time*group_ = 0.98, *p* = 0.45, Partial *η2* = 0.21), but the time term showed a significant difference in the heart rate recovery rate between the A curve and the A _linear_ with time point (*df* = 4, *f*_time_ = 13.82, *p* < 0.001, Partial *η2* = 0.79). LSD post hoc tests showed a decreasing trend in A _curved_ between the first minute (*M* ± *SD* = 90.60 ± 14.42), second minute (*M* ± *SD* = 83.05 ± 14.73), third minute (*M* ± *SD* = 81.80 ± 15.44), fourth minute (*M* ± *SD* = 79.40 ± 12.53) and fifth minute (*M* ± *SD* = 80.50 ± 12.05). The difference of mean and the geometric trend shows that the participants’ heart rate recovered more quickly between 1 and 3 min and gradually decreased between 4 and 5 min. Similarly, in the A _linear_, the first minute (*M* ± *SD* = 95.90 ± 12.62), second minute (*M* ± *SD* = 86.85 ± 12.22), third minute (*M* ± *SD* = 85.85 ± 11.58), fourth minute (*M* ± *SD* = 86.05 ± 12.76) and fifth minute (*M* ± *SD* = 85.40 ± 11.62) indicated a decreasing trend in the participants’ recovery rate from 1 to 5 min. Recovery was faster from 1 to 2 min and slower from 3 to 5 min.

Furthermore, we investigated the interaction of continuous measurements data with time in environment B. The Mauchly’s sphericity test indicated that data of environment B do not meet the spherical hypothesis (*p* < 0.001). The time*group term in the multivariate test was statistically significant (*df* = 4, *f*_time*group_ = 3.40, *p* = 0.04, Partial *η2* = 0.48). The time term showed that the heart rate recovery rates of the B _curved_ and B _linear_ also had significant differences with time point (*df* = 4, *f*_time_ = 7.41, *p* = 0.002, Partial *η2* = 0.66). LSD post hoc tests showed that the differences in recovery between each other at the first (*M* ± *SD* = 86.40 ± 11.46), second (*M* ± *SD* = 79.25 ± 14.08), third (*M* ± *SD* = 79.25 ± 15.46), fourth (*M* ± *SD* = 78.70 ± 13.54) and fifth minutes (*M* ± *SD* = 78.10 ± 14.37) were significant. There was a decreasing trend in the participants’ recovery effects from 1 to 5 min, with faster recovery at 1–2 min and slower at 3–5 min. The recovery trend for B _linear_ is similar with B _curved_, with decreasing trends in the first (*M* ± *SD* = 100.95 ± 14.46), second (*M* ± *SD* = 92.05 ± 13.67), third (*M* ± *SD* = 88.20 ± 14.20), fourth (*M* ± *SD* = 87.90 ± 13.99), and fifth minutes (*M* ± *SD* = 90.10 ± 13.70).

## 6. Discussion

### 6.1. The Effect of Environmental Geometry on Human Stress Recovery

In this research, we investigated the recovery effects of linear and curved environments in transitional spaces on human stress. Forty participants were randomly assigned to enter one of four (split into two groups) virtual transitional spaces, i.e., the linear plaza space, the curved plaza space, the linear café, and the curved café. We compared the ∆D for HR, STAI and BP after the validation of the experimental randomization. The experimental results strongly support our first hypothesis that linear and curvilinear environments in transition spaces have different effects on human stress recovery. The data suggest that all four environments have a positive effect on human heart rate, STAI and blood pressure recovery. However, overall, in this experiment, curved spaces have a more positive effect on human stress recovery compared to linear spaces (i.e., A _curved_ has a better pressure recovery rate than A _linear_; B _curved_ has a better pressure recovery rate than B _linear_). Meanwhile, we have not yet found a negative effect of linear and curved environments on human stress recovery. This suggests that the appropriate use of curved elements in transitional spaces can contribute to the relief of human stress. Previous research has demonstrated that the curved environment has a more positive effect on human emotions than the linear environment in indoor and outdoor spaces [26,32,62]. The results of this research agree, refine and develop previous findings, that is, more curved factors in indoor and outdoor spaces have a better effect on the recovery of human stress [26,27,28,32,36,37,62]. We propose a new type of space apart from indoor and outdoor spaces—the transitional space—and provide evidence for the effect of transitional spaces on human emotions.

### 6.2. Comparison of Psychological and Physiological Data Results

The results of this study indicated that the participants showed better recovery from stress on both psychological and physical measures, with statistically significant results. However, the participants generally had smaller effect size for the psychological data compared to the physiological data. The effect size of the STAI score, which reflects psychological indicators, was small (A_environment_ Cohen’s *d* = 0.38) and moderate (B_environment_ Cohen’s *d* = 0.78), while the effect sizes of SBP, DBP and HR, which reflect physiological indicators, were mostly large (Cohen’s *d* > 0.8). This means that some changes that the participants were not aware of psychologically were reflected physiologically. We speculate that this is possibly due to the short duration of the experiment. The questionnaires do not provide as much immediate feedback as physiological responses. This provides a reference for future studies in short-term laboratory environments, where the physiological data of the participants is a more obvious reflection of changes in emotion or stress than a questionnaire.

### 6.3. Potential for Transitional Spaces to Improve Population Stress

To investigate the relationship between recovery effects in transition space and time, we analyzed the interaction effect between continuous measurement data and time. The repeated measures ANOVA showed that during recovery, participants typically recovered more efficiently between 1 and 3 min, with a gradual decrease in recovery efficiency between 4 and 5 min. The geometric recovery rate of the physiological data proves that the transition space is able to recover approximately 70% of human surge in stress at 1–3 min. Due to the nature of transitional spaces, people do not usually stay for long periods in transitional spaces compared to indoor and outdoor spaces. Therefore, this suggests that we should focus more on the short-term effects of transitional spaces on human stress recovery than on the long-term effects. The experimental results indicated that transitional spaces could recover human stress in the short term and, with appropriate spatial arrangements, would potentially contribute to reduce the stress of human in contemporary urban spaces.

## 7. Limitations and Future Research

While this study was successful in testing the hypothesis, there are still some limitations to this research. Some scholars have a negative perception of the use of VR in environmental research, arguing that VR is not a substitute for real-world scenarios [63,64]. Some scholars also believe that the stress recovery process is related to a variety of factors, such as hearing, smell, light perception, and thermal comfort. [48,65]. However, VR as a new research tool can isolate the effect of specific factors on the outcome, controlling the complex factors that affect the outcome into a single factor, which may not be possible in the real world. There are also some research studies that suggest that the experimental effects of VR scenes and real-world scenes are the same [66,67,68]. Furthermore, more in-depth research on the effect of transitional spaces on human stress would be more useful in guiding practical planning and design. This study only investigated the effects of transitional spaces on human stress recovery in four scenarios, although these four scenarios were representative of transitional spaces. Other types of transitional spaces can be researched in the future. We intend to implement our findings into studying other transitional spaces such as staircases, corridors, foyers, and lift lobbies. In addition, this study did not control for curve rate or “curviness” in a curved environment; future research could control for different curve rates in the environment and investigate the curve rate in transitional space that has the best effect on human stress recovery. This study measured the participants’ heart rate, diastolic blood pressure, systolic blood pressure and STAI, and future studies could include other measurement devices such as electroencephalogram (EEG) to analyze the effects on human stress from a neuroscientific perspective. The exposure time for this research was 40 min due to the laboratory method, and the changes in the participants’ psychological questionnaires were not enough to observe. In future studies, we will consider long-term observations of the participants’ stress changes. 

## 8. Conclusions

The study investigated the effects of curved and linear environments in transitional spaces on human stress recovery through subjective questionnaires and monitoring of physiological variables in the laboratory. It provided some evidence that the geometry of the environment in transitional spaces can reduce human stress to achieve human wellbeing. There are three main findings of this research. First, in the transition space, the curved environment had a better stress recovery effect on the participants than the linear environment. Second, in this experiment, the participants showed significant effect size for both the subjective questionnaire and the physiological variables. Furthermore, the physiological parameters showed larger effect size than the subjective questionnaire, indicating that the participants’ physiological responses were more significant than their psychological responses. Third, time interacted with physiological parameters, with the participants usually having recovered 70% of their pressure in the first 1–3 min and gradually decreasing in 4–5 min. As a type of space that is frequently visited in the contemporary city context, a transitional space could potentially become an urban shelter that is revitalizing, alleviating the stressful impact of increasing crowding. With that in mind, we could envision the near future with optimism that designers, developers and government officials could acknowledge the unique features of the curved architectural elements and therefore facilitate more stress-recovery friendly transitional spaces for the wellbeing of the broader community. 

## Figures and Tables

**Table 1:** Participant personal information.

Title 1	Overall	A _Linear_	A _Curved_	B _Linear_	B _Curved_
	Mean ± SD or n (%)				
Number of Participant	40	10	10	10	10
Age	25 ± 4	25 ± 4	24 ± 4	24 ± 4	25 ± 3
Gender	--	--	--	--	--
Female	20 (50)	5 (50)	5 (50)	5 (50)	5 (50)
Male	20 (50)	5 (50)	5 (50)	5 (50)	5 (50)

**Table 2:** Test on whether participants’ physical data and psychological data at baseline (pre-TSST), post-TSST and IEQ among four environments were similar or not.

Measures	Method	F	df	*p* Value
Pre-TSST				
Heart Rate (bmp)	ANOVA	0.86	3	0.47
Systolic Blood Pressure (mmHg)	ANOVA	0.63	3	0.60
Diastolic Blood Pressure(mmHg)	ANOVA	0.86	3	0.47
Post-TSST				
Heart Rate (bmp)	ANOVA	0.62	3	0.61
Systolic Blood Pressure (mmHg)	ANOVA	0.04	3	0.99
Diastolic Blood Pressure(mmHg)	ANOVA	0.61	3	0.61
State-Anxiety Inventory	ANOVA	0.81	3	0.49
IEQ				
CO_2_ (ppm)	ANOVA	0.55	3	0.66
PM_2.5_ (µg/m^3^)	ANOVA	0.48	3	0.70
Temperature (°C)	ANOVA	1.57	3	0.25
Humidity (%)	ANOVA	0.77	3	0.53

**Table 3:** Test on whether 10 min of TSST produced significant stress on participants.

Measures	Method	T	df	*p* Value
Heart Rate (bmp)	Paired *t* test	−15.59	39	<0.001
Systolic Blood Pressure (mmHg)	Paired *t* test	−3.54	39	0.001
Diastolic Blood Pressure (mmHg)	Paired *t* test	−3.14	39	0.003

## Data Availability

The research data is not publicly available due to privacy constraints.
